# Impostor phenomenon among Saudi university students: associations with maladaptive perfectionism, self-efficacy, and psychological well-being

**DOI:** 10.3389/fpsyg.2026.1888808

**Published:** 2026-07-17

**Authors:** Fahad Alzahrani, Khadijah A. Aytah, Alaa M. Alhammad, Seba M. Alshehri, Shoug Alhinti, Shifaa S. Alyoubi, Abdulrahman M. Alhammad, Ibrahim Mohammad Bassati, Haifa A. Fadil, Ahmed Aldhafiri, Faris S. Alnezary

**Affiliations:** 1 Department of Pharmacy Practice, College of Pharmacy, Taibah University, Madinah, Saudi Arabia; 2 Scientific Research Unit, College of Pharmacy, Taibah University, Madinah, Saudi Arabia; 3 Pharmaceutical Analysis Department, King Abdullah International Medical Research Center, Riyadh, Saudi Arabia; 4 Pharmacology and Toxicology Department, College of Pharmacy, Taibah University, Madinah, Saudi Arabia

**Keywords:** cross-sectional study, happiness, impostor phenomenon, maladaptive perfectionism, Saudi Arabia, self-efficacy, sense of belonging, university students

## Abstract

**Background:**

The impostor phenomenon (IP) is characterized by persistent self-doubt and fears of being exposed as intellectually inadequate despite evidence of competence. Although IP has received increasing international attention, comparatively few studies have simultaneously examined its associations with maladaptive perfectionism, self-efficacy, belonging, and wellbeing within Middle Eastern university contexts. This study examined the associations among IP, maladaptive perfectionism, self-efficacy, happiness, sense of belonging, and perceived academic competition among university students in Saudi Arabia.

**Methods:**

A cross-sectional study was conducted among 505 undergraduate students at Taibah University, Saudi Arabia. Participants completed validated measures assessing IP, self-efficacy, maladaptive perfectionism, happiness, belonging, and perceived academic competition. Inferential statistics and bootstrapped indirect association analyses using Hayes' PROCESS Model 4 were conducted.

**Results:**

IP demonstrated a moderate positive association with maladaptive perfectionism (r = 0.56, p < 0.01) and weak negative associations with self-efficacy (r = −0.19, p < 0.01) and happiness (r = −0.20, p < 0.01). Self-efficacy showed moderate positive associations with happiness (r = 0.53, p < 0.01) and sense of belonging (r = 0.40, p < 0.01). Indirect association analyses identified statistically significant patterns of association among maladaptive perfectionism, IP, and self-efficacy. Students from administrative and humanities colleges reported higher IP scores than students from health and scientific colleges.

**Conclusion:**

IP among Saudi university students was associated with greater maladaptive perfectionism and lower self-efficacy, whereas self-efficacy demonstrated stronger positive associations with happiness and belonging. The findings highlight the importance of cognitive and emotional factors associated with students' psychological experiences in higher education settings. Given the cross-sectional design, all findings should be interpreted cautiously, and longitudinal studies are needed to better understand these relationships over time.

## Introduction

The Impostor Phenomenon (IP) is defined as a persistent internal experience of intellectual fraudulence and self-doubt, characterized by an inability to internalize personal achievements despite objective evidence of competence (Clance and Imes, 1978; King and Cooley, 1995). Individuals experiencing IP often attribute their success to external factors such as luck, timing, or interpersonal skills while maintaining a persistent fear of being exposed as “frauds.” Although early research primarily focused on high-achieving women, contemporary literature recognizes IP as a broader psychological concern affecting diverse populations, particularly in higher education settings (Clance and Imes, 1978). Academic environments characterized by continuous evaluation and social comparison may contribute to impostor experiences and have been associated with anxiety, depression, burnout, and reduced psychological wellbeing (Bravata et al., 2020; Parkman, 2016; Weir, 2013).

The present study is grounded in Social Cognitive Theory (SCT), which proposes that human functioning reflects reciprocal interactions among cognitive, behavioral, and environmental factors. Within this framework, self-efficacy, defined as individuals' beliefs in their ability to successfully perform specific actions, is considered an important component of emotional and behavioral functioning (Shinawatra et al., 2023). Students with lower self-efficacy may report stronger impostor feelings because they may be less likely to attribute academic success to their own abilities (Bandura, 1997; Cozzarelli and Major, 1990). In addition, maladaptive perfectionism, characterized by excessive self-criticism and concern over mistakes, has frequently been associated with impostor experiences (Pákozdy et al., 2024). These perfectionistic tendencies may be associated with greater fear of failure, lower wellbeing, and reduced sense of belonging within academic settings (Sakulku, 2011). Conversely, stronger self-efficacy may support academic engagement and social integration (Shinawatra et al., 2023).

Although research on the IP has increased internationally and within Saudi Arabia, existing studies have largely focused on prevalence estimates or isolated associations with academic performance, burnout, and psychological distress (Clance, 1986; El-Setouhy et al., 2024). Consequently, there remains a limited understanding of how IP relates simultaneously to maladaptive perfectionism, self-efficacy, happiness, sense of belonging, and perceived academic competition within an integrated analytical framework. This gap is particularly important in Saudi Arabia, where higher education has undergone substantial expansion and increasing academic competitiveness, potentially influencing students‘ achievement-related self-perceptions and psychological wellbeing (Alrayyes et al., 2020). By examining these interrelated psychological constructs concurrently, the present study extends previous literature beyond single-variable investigations and provides a more comprehensive understanding of the psychological associations surrounding IP among university students in a Middle Eastern context. Such understanding may help inform interventions to promote students' psychological wellbeing and reduce distress associated with impostor experiences.

The present study aimed to examine the associations between IP, maladaptive perfectionism, self-efficacy, sense of belonging, and happiness among Saudi university students. Based on SCT and previous literature, the study explored whether: (1) maladaptive perfectionism was statistically associated with the relationship between IP and self-efficacy; (2) IP was statistically associated with the relationship between self-efficacy and sense of belonging; and (3) IP and self-efficacy were statistically associated with the relationship between perfectionism and happiness.

## Methods

### Study design and study population

A cross-sectional study was conducted among undergraduate students from various colleges at Taibah University in Saudi Arabia, between September and December, 2025. A combination of convenience and snowball sampling techniques was used to recruit participants. Initial recruitment occurred through official university WhatsApp groups using a digital survey developed via Google Forms. Participants were encouraged to share the survey within their academic networks to enhance recruitment. Inclusion criteria were: (1) enrollment as an undergraduate student at Taibah University, (2) age ≥18 years, and (3) ability to understand Arabic. Responses with substantial missing data were excluded from analysis.

While this approach facilitated access to a large sample, it may increase the risk of selection bias and limit representativeness. Additionally, as the study was conducted within a single institution, the generalizability of the findings to other universities may be constrained.

Taibah University is one of the largest universities in Saudi Arabia, with an enrollment of approximately 57,535 students, 61% of whom are female (Chua et al., 2025). A priori sample size estimation was conducted using G^*^Power (α = 0.05, power = 0.95), indicating a minimum required sample of 383 participants. The final sample of 505 students exceeded this requirement, indicating adequate statistical power for the planned indirect association analyses (Council of Universities' Affairs GSUaSS, 2025).

## Measures

The questionnaire was conducted in Arabic and included established psychometric tools commonly used in previous studies. These instruments were not created by the researchers themselves. When validated Arabic versions were not accessible, standard translation methods, such as independent forward translation, back-translation, and expert review, were employed to guarantee linguistic and cultural accuracy.

### Impostor phenomenon

The Clance Impostor Phenomenon Scale (CIPS) was used to assess impostor experiences. The scale consists of 20 items rated on a 5-point Likert scale ranging from 1 (“Never”) to 5 (“Very often”), with total scores ranging from 20 to 100. Higher scores indicate greater impostor experiences (Sim et al., 2022; Clance, 1985). The CIPS demonstrated good to excellent reliability in previous studies (Cozzarelli and Major, 1990; Simon et al., 2018) and good internal consistency in the present study (Cronbach's α = 0.89).

### Self-efficacy

Self-efficacy was assessed using the New General Self-Efficacy Scale (NGSE), which consists of 8 items rated on a 5-point Likert scale ranging from 1 (“Strongly disagree”) to 5 (“Strongly agree”). Total scores range from 8 to 40, with higher scores indicating greater perceived self-efficacy (French et al., 2008). The NGSE demonstrated good to excellent reliability in previous studies (Cozzarelli and Major, 1990; Chen et al., 2001) and excellent internal consistency in the present study (Cronbach's α = 0.92).

### Maladaptive perfectionism

Maladaptive perfectionism was assessed using the self-critical perfectionism dimension of the Big Three Perfectionism Scale–Short Form (BTPS-SF), a multidimensional measure of perfectionism that includes rigid, self-critical, and narcissistic dimensions (Scherbaum et al., 2006). The self-critical dimension of the BTPS-SF was used to operationalize maladaptive perfectionism because it reflects excessive self-criticism, concern over mistakes, and negative self-evaluation, which are theoretically associated with impostor experience (Feher et al., 2020). Items were rated on a 5-point Likert scale, with higher scores indicating greater maladaptive perfectionism. The BTPS-SF demonstrated good to excellent reliability in previous studies (Cozzarelli and Major, 1990; Alammar et al., 2025) and excellent internal consistency in the present study (Cronbach's α = 0.92).

### Happiness

The Oxford Happiness Questionnaire (OHQ) was used to assess subjective wellbeing. The scale consists of 8 items rated on a 5-point Likert scale, with higher scores indicating greater happiness (Kaçar-Başaran et al., 2022). The OHQ demonstrated satisfactory psychometric properties in previous studies (Cozzarelli and Major, 1990; Hills and Argyle, 2002) and acceptable internal consistency in the present study (Cronbach's α = 0.76).

### Belonging

Sense of belonging was measured using a five-item social identification scale assessing ingroup ties, centrality, and ingroup affect (Özdemir, 2020). Responses were rated on a 5-point Likert scale, with higher scores indicating stronger perceived belonging. The sense of belonging scale demonstrated satisfactory reliability in a previous study (Cozzarelli and Major, 1990). The scale demonstrated good internal consistency in the present study (Cronbach's α = 0.73).

### Perceived academic competition

Perceived academic competition was assessed using a two-item scale adapted for the academic context (Cameron, 2004). Responses were rated on a 5-point Likert scale, with higher scores indicating greater perceived academic competition. The perceived academic competition scale demonstrated satisfactory reliability in a previous study (Cozzarelli and Major, 1990) and acceptable internal consistency in the present study (Cronbach's α = 0.72).

## Data Collection Procedures

Participants completed the online questionnaire in Arabic after providing informed consent. The psychometric measures (CIPS, NGSE, BTPS-SF, and OHQ) were administered in a standardized sequence, followed by demographic questions. Upon completing the survey, participants received a debriefing statement and were entered into a voluntary prize draw, in which five participants received 100 Saudi Riyals. Participation in the prize draw was not contingent on specific responses.

Before data collection, the instrument was evaluated by three experts in psychology and public health for content relevance and clarity, and then pilot tested with eight students from the target population to assess comprehensibility and face validity. Minor wording adjustments were made in response to the feedback received. A test–retest procedure was also conducted with 24 students over a 1 week interval to evaluate score stability. The test–retest reliability coefficient was 0.96, indicating excellent temporal stability and exceeding the predefined acceptable threshold of 0.80 (Canning, 2020).

## Data analysis

Statistical analyses were conducted using IBM SPSS Statistics (Version 27). Prior to analysis, reverse-coded items were recoded, and total scores for each scale were computed. Descriptive statistics, including means and standard deviations, were calculated for all study variables. Independent-samples *t*-tests were used to compare differences between two groups, while one-way analysis of variance (ANOVA) was used for comparisons involving more than two groups. Pearson correlation coefficients were calculated to examine associations among the study variables.

Indirect association analyses were conducted using Model 4 of Hayes's PROCESS macro (version 3.3). Although PROCESS is commonly used to estimate indirect effects, the present study's cross-sectional design does not permit causal inference. Accordingly, the analyses were interpreted as patterns of statistical association rather than evidence of causal mediation. Three models were examined: (1) maladaptive perfectionism in the association between IP and self-efficacy, (2) IP in the association between self-efficacy and sense of belonging, and (3) IP and self-efficacy in the association between maladaptive perfectionism and happiness. All variables were standardized prior to analysis.

Bootstrapping with 5,000 resamples and 95% confidence intervals (CI) was used to estimate indirect effects. Indirect associations were considered statistically significant when the CI did not include zero. Bootstrapping procedures were selected because they provide robust estimates of indirect effects and are less sensitive to violations of normality assumptions. Given the cross-sectional design, all mediation findings were interpreted as statistical associations rather than causal relationships (Council of Universities' Affairs GSUaSS, 2025; Shawahna et al., 2017).

## Results

### Demographic characteristics

The sample consisted of 505 undergraduate students, the majority of whom were female (84.4%), with most participants aged 18-20 years (51.3%). The mean impostor phenomenon (IP) score was numerically higher among females (*M* = 3.41) compared to males (*M* = 3.34), although this difference was not statistically significant (*p* = 0.43).

No statistically significant differences in IP scores were observed across age groups (*p* = 0.24), academic level (*p* = 0.25), or GPA categories (*p* = 0.69). However, a statistically significant difference was observed across academic disciplines (p = 0.03), with students from administrative and humanities colleges reporting higher IP scores than those in health and scientific colleges (Table 1).

**Table 1:** Students' demographic characteristics.

Characteristics	Category	*n*	%	Mean IP score ±SD	*p value*
Gender	Male	79.0	15.6	3.34 ± 0.08	0.43
	Female	426.0	84.4	3.41 ± 0.03	
Age	18-20	259	51.3	3.46 ± 0.04	0.24
	21-23	209	41.4	3.35 ± 0.05	
	24-26	27	5.3	3.40 ± 0.14	
	27-29	10	2.0	3.21 ± 0.30	
College	Health colleges	253	50.0	3.35 ± 0.04	0.03^*^
	Scientific colleges	139	27.5	3.37 ± 0.06	
	Administrative and Humanities Colleges	113	22.4	3.56 ± 0.40	
Academic level	First	68	13.5	3.57 ± 0.08	0.25
	Second	108	21.4	3.43 ± 0.06	
	Third	155	30.7	3.41 ± 0.05	
	Fourth	98	19.4	3.36 ± 0.07	
	Fifth	41	8.1	3.23 ± 0.10	
	≥6	35	6.9	3.27 ± 0.15	
GPA (out of 5)	< 3	18	3.6	3.26 ± 0.35	0.69
	3.00-3.49	37	7.3	3.37 ± 0.13	
	3.50-3.99	67	13.3	3.45 ± 0.09	
	4.00-4.49	152	30.1	3.37 ± 0.06	
	4.50-5.00	185	36.6	3.43 ± 0.05	
	Nothing (first year)	45	8.9	3.39 ± 0.11	

^**^p < 0.01; ^*^p < 0.05.

### Correlation analysis

Pearson correlation analysis revealed several statistically significant associations among the study variables (Table 2). A moderate positive association was observed between impostor phenomenon (IP) and maladaptive perfectionism (*r* = 0.56, *p* < 0.001). In contrast, weak negative associations were identified between IP and self-efficacy (*r* = −0.19, *p* < 0.001) and between IP and happiness (*r* = −0.20, *p* < 0.001). IP also showed a weak positive association with sense of belonging (*r* = 0.09, *p* < 0.05), while no statistically significant association was observed between IP and perceived academic competition (*r* = 0.01, *p* > 0.05).

**Table 2:** Mean, standard deviation, and correlation of main variables (*n* = 505).

	*M*	*SD*	1	2	3	4	5	6
1. CIPS	2.93	0.76		−0.19^**^	0.56^**^	−0.20^**^	0.09^*^	0.01
2. NGSE	3.81	0.82	−0.19^**^		0.03	0.53^**^	0.40^**^	0.30^**^
3. BTPS-SF	2.97	0.74	0.56^**^	0.03		−0.07	0.23^**^	0.10^*^
4. OHQ	3.49	0.53	−0.20^**^	0.53^**^	−0.07		0.35^**^	0.28^**^
5. Belonging	3.67	0.84	0.09^*^	0.40^**^	0.23^**^	0.35^**^		0.85^**^
6. Competitiveness	3.64	1.07	0.01	0.30^**^	0.10^*^	0.28^**^	0.85^**^

Clance Impostor Phenomenon Scale (CIPS), New General Self-Efficacy Scale (NGSE), Big Three PerfectionismScale-Short Form (BTPS-SF), and Oxford Happiness Questionnaire (OHQ). ^*^p < 0.05 (2-tailed); ^**^p < 0.01(2-tailed).

Self-efficacy demonstrated moderate positive associations with happiness (r = 0.53, *p* < 0.001), sense of belonging (*r* = 0.40, *p* < 0.001), and perceived academic competition (*r* = 0.30, *p* < 0.01). Maladaptive perfectionism showed weak positive associations with belonging (*r* = 0.23, *p* < 0.01) and competitiveness (*r* = 0.10, *p* < 0.05), while its association with happiness was not statistically significant (*r* = −0.07, *p* > 0.05).

A strong positive association was observed between sense of belonging and perceived academic competition (r = 0.85, p < 0.001), suggesting substantial overlap between these constructs within the present sample. Overall, the findings suggest stronger associations between IP and maladaptive perfectionism than between IP and self-efficacy or happiness.

Clance Impostor Phenomenon Scale (CIPS), New General Self-Efficacy Scale (NGSE), Big Three Perfectionism Scale—Short Form (BTPS-SF), and Oxford Happiness Questionnaire (OHQ). ^*^p < 0.01 (2-tailed); *p* < 0.05 (2-tailed).

### Indirect association analyses

Three indirect association models were examined using Hayes' PROCESS macro (Model 4). The findings are presented as patterns of statistical association rather than evidence of causal relationships.

*Model 1. Indirect Association Pattern Involving Impostor Phenomenon, Maladaptive Perfectionism, and Self-Efficacy*.

**Figure 1:**
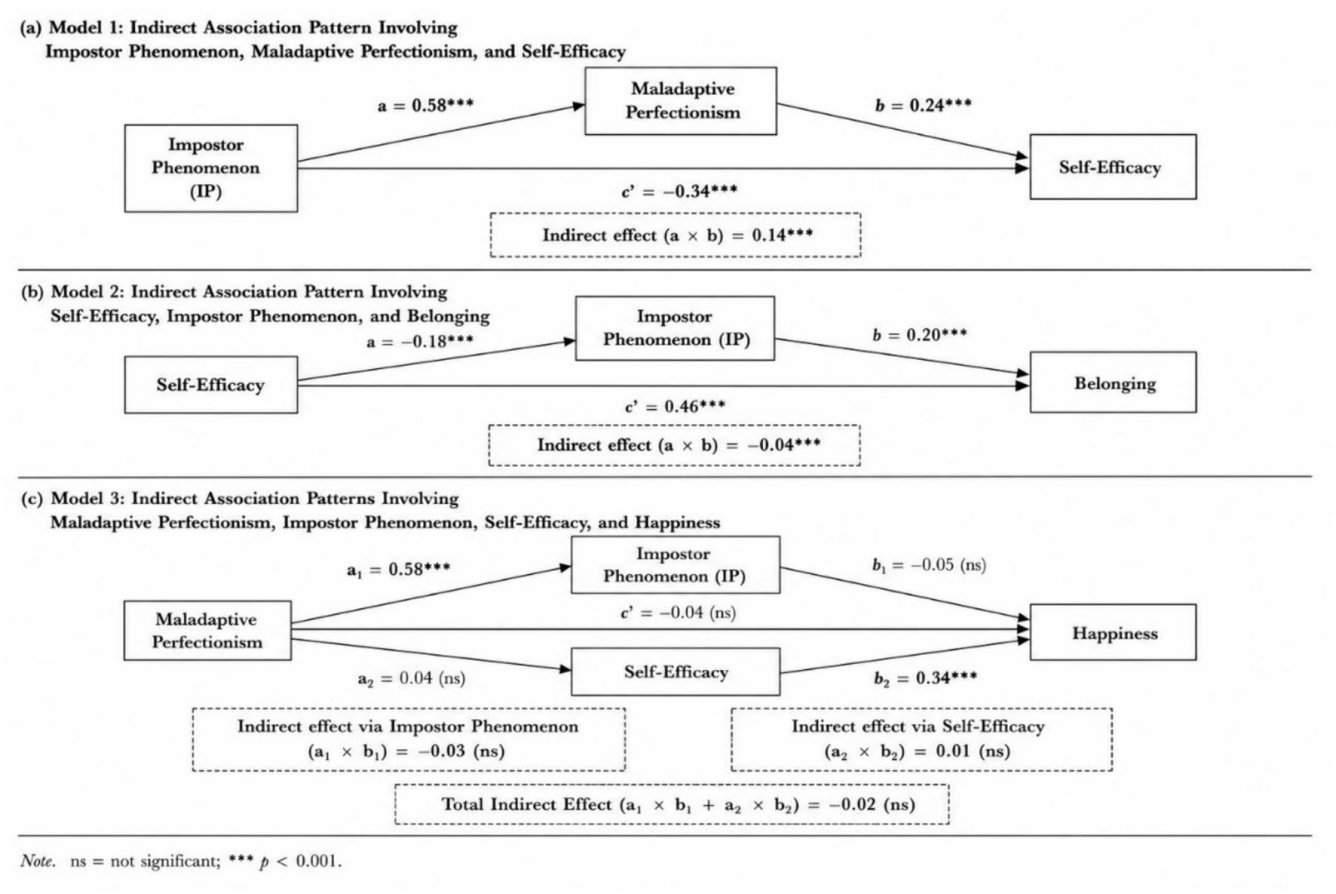
Indirect association models illustrating relationships among impostor phenomenon, maladaptive perfectionism, self-efficacy, belonging, and happiness. Standardized coefficients are presented. **(a)**. Indirect association pattern involving impostor phenomenon, maladaptive perfectionism, and self- efficiency. **(b)** Model 2. Indirect association pattern involving self- efficiency, impostor phenomenon and belonging. **(c)** Model 3. Indirect association pattern involving maladaptive perfectionism, impostor phenomenon, and self-efficiency, and happiness. Note: ns = non significant; ***p < 0.001.

IP was positively associated with maladaptive perfectionism (B = 0.55, *p* < 0.01), while maladaptive perfectionism was positively associated with self-efficacy (*B* = 0.23, *p* < 0.01). The total association between IP and self-efficacy was negative (B = −0.20, *p* < 0.01), and the direct association remained negative after including maladaptive perfectionism in the model (B = −0.33, *p* < 0.01). These findings indicate an indirect association pattern involving impostor phenomenon, maladaptive perfectionism, and self-efficacy (Table 3, Figure 1a).

**Table 3:** Indirect Association Analyses (*N* = 505).

Path	B	SE	t	*p value*	95% CI (LL – UL)	Std. Coeff.
Model 1: Indirect association pattern involving impostor phenomenon, maladaptive perfectionism, and self-efficacy						
a: CIPS → BTPS-SF	0.55	0.03	15.46	< 0.001	[0.48, 0.62]	0.56
b: BTPS-SF → NGSE	0.23	0.05	4.16	< 0.001	[0.12, 0.35]	0.21
c: CIPS → NGSE (Total)	−0.20	0.04	−4.40	< 0.001	[−0.29, −0.11]	−0.19
c′: CIPS → NGSE (Direct)	−0.33	0.05	−6.04	< 0.001	[−0.44, −0.22]	−0.31
Model 2: Indirect association pattern involving self-efficacy, impostor phenomenon, and belonging						
a: NGSE → IP	−0.17	0.04	−4.4	< 0.001	[−0.25, −0.09]	−0.19
b: CIPS → Belonging	0.20	0.04	4.4	< 0.001	[0.11, 0.29]	0.18
c: NGSE → Belonging (Total)	0.42	0.04	9.98	< 0.001	[0.33, 0.50]	0.40
c′: NGSE → Belonging (Direct)	0.45	0.04	10.84	< 0.001	[0.37, 0.53]	0.44
Model 3: Indirect association patterns involving maladaptive perfectionism, impostor phenomenon, self-efficacy, and happiness						
a1: BTPS-SF → CIPS	0.58	0.03	15.46	< 0.001	[0.50, 0.65]	0.56
a2: BTPS-SF → NGSE	0.04	0.05	0.85	0.40	[−0.05, 0.13]	0.03
b1: CIPS → OHQ	−0.05	0.03	−1.58	0.11	[−0.11, 0.01]	−0.07
b2: NGSE → OHQ	0.34	0.02	13.34	< 0.001	[0.29, 0.39]	0.51
c: BTPS-SF → OHQ (Total)	−0.05	0.03	−1.75	0.08	[−0.11, 0.00]	−0.07
c′: BTPS-SF → OHQ (Direct)	−0.04	0.03	−1.19	0.23	[−0.10, 0.02]	0.05

Clance Impostor Phenomenon Scale (CIPS), New General Self-Efficacy Scale (NGSE), Big Three Perfectionism Scale—Short Form (BTPS-SF), and Oxford Happiness Questionnaire (OHQ).

*Model 2. Indirect Association Pattern Involving IP, Self-Efficacy, and Belonging*.

Self-efficacy was negatively associated with IP (B = −0.17, *p* < 0.01), whereas IP was weakly positively associated with belonging (B = 0.20, *p* < 0.01). The total association between self-efficacy and belonging was positive (B = 0.42, *p* < 0.01), and the direct association remained positive after IP was included in the model (B = 0.45, p < 0.01). The indirect effect was negative (B = −0.04, *p* < 0.01), whereas the total and direct associations were positive, indicating an inconsistent pattern of indirect associations involving self-efficacy, IP, and belonging (Table 3, Figure 1b).

*Model 3. Indirect Association Patterns Involving Maladaptive Perfectionism, Impostor Phenomenon, Self-Efficacy, and Happiness*

Perfectionism was positively associated with IP (B = 0.58, *p* < 0.001), whereas its association with self-efficacy was not statistically significant (B = 0.04, *p* > 0.05). IP was not significantly associated with happiness (B = −0.05, *p* > 0.05), while self-efficacy was positively associated with happiness (B = 0.34, *p* < 0.001). The direct association between perfectionism and happiness was not statistically significant (B = −0.04, *p* > 0.05). Neither the indirect effect via IP (a1 × b1 = −0.03, *p* > 0.05) nor the indirect effect via self-efficacy (a2 × b2 = 0.01, *p* > 0.05) reached statistical significance. The total indirect effect was also not statistically significant (a1 × b1 + a2 × b2 = −0.02, *p* > 0.05) (Table 3, Figure 1c).

## Discussion

This study examined the psychological dynamics of the impostor phenomenon (IP) among Saudi university students, with a particular focus on its associations with maladaptive perfectionism, self-efficacy, happiness, sense of belonging, and perceived academic competition. Overall, the findings highlight several statistically significant relationships that help explain how IP is linked to key psychological constructs in the higher education context.

One notable finding was variation in IP levels across academic disciplines, with students from Administrative and humanitarian colleges reporting higher scores than those in health and scientific fields. This pattern contrasts with prior research that often reports elevated IP in science and health-related disciplines (Hayes, 2017). Similar associations between impostor experiences, perfectionism, and self-efficacy have also been reported among university students in Europe, North America, and Russia, suggesting that these psychological processes may extend across diverse educational and cultural settings (Cozzarelli and Major, 1990; Chakraverty, 2020). This discrepancy may reflect contextual differences in perceived career uncertainty, job market competitiveness, or academic support structures across disciplines. These findings suggest that institutional and environmental factors may shape the expression of impostor experiences in different academic contexts.

The correlation analysis revealed a moderate positive association between IP and maladaptive perfectionism, as well as weak negative associations between IP and both self-efficacy and happiness. These findings are consistent with previous literature suggesting that individuals experiencing impostor feelings may engage in self-critical perfectionistic tendencies as a way of coping with perceived inadequacy (Cozzarelli and Major, 1990). Importantly, the relatively weak associations between IP, self-efficacy, and happiness indicate that these relationships are present but modest, highlighting the complexity of the observed psychological associations.

An unexpected finding was the very strong positive association between sense of belonging and perceived academic competition. Although these constructs are theoretically distinct, they may be closely intertwined in highly competitive educational environments (Cameron, 2004). Students who perceive themselves as integrated and connected within their academic community may simultaneously become more aware of peer comparison and achievement-related competition. Nevertheless, the magnitude of this association suggests substantial conceptual overlap within the present sample and should therefore be interpreted cautiously.

From a theoretical perspective, these findings align with Social Cognitive Theory, which emphasizes the central role of self-efficacy in shaping emotional and cognitive responses (Shinawatra et al., 2023). Within this framework, IP may reflect a distortion in self-appraisal processes, in which individuals underestimate their abilities despite objective evidence of competence (Sheveleva et al., 2023). This distorted self-perception may be associated with lower self-efficacy and reduced wellbeing, although the strength of these associations appears limited in the present study (Cozzarelli and Major, 1990; Vergauwe et al., 2015).

The first indirect association model identified statistically significant associations involving maladaptive perfectionism in the relationship between IP and self-efficacy. Although IP was negatively associated with self-efficacy overall, maladaptive perfectionism was positively associated with self-efficacy within the statistical model. This finding highlights the complexity and multidimensional nature of perfectionism, as perfectionistic tendencies may involve both achievement-oriented striving and self-critical evaluation (Clance and O'Toole, 2014; Lo and Abbott, 2019).

The second model demonstrated an indirect association pattern involving self-efficacy, IP, and sense of belonging. Students with higher self-efficacy reported greater belonging, consistent with previous research linking self-efficacy to engagement and social integration in academic settings (Shafran and Mansell, 2001; Hoffman et al., 2002; Pittman and Richmond, 2007). Self-efficacy was also negatively associated with IP, indicating that lower self-efficacy was associated with stronger impostor feelings. Although IP was positively associated with belonging, the magnitude of this association was relatively weak. Moreover, the indirect effect was negative, whereas the total and direct associations between self-efficacy and belonging were positive, indicating an inconsistent suppression-like pattern of indirect associations that should be interpreted with caution (Freeman et al., 2007).

The third model did not identify statistically significant indirect associations involving IP or self-efficacy in the relationship between perfectionism and happiness. While perfectionism was strongly associated with IP, neither perfectionism nor IP showed a statistically significant direct association with happiness in the model, consistent with work suggesting that perfectionistic tendencies may be associated with wellbeing through related cognitive and affective factors rather than direct associations (Chakraverty, 2020). In contrast, self-efficacy demonstrated a strong positive association with happiness, aligning with prior evidence that self-efficacy is positively related to life satisfaction and subjective happiness and can act as a mediator linking personality characteristics to wellbeing (Kennedy, 2024). These findings highlight the strong positive association between self-efficacy and happiness within the present sample and are consistent with previous research identifying self-efficacy as an important factor associated with perfectionism and psychological wellbeing (Strobel et al., 2011; Kruger et al., 2023).

Previous literature has also conceptualized IP through emotional processes, particularly the distinction between shame and guilt. Shame involves negative evaluations of the self as a whole, whereas guilt is focused on specific behaviors (Sahin, 2021). IP has been more closely associated with shame-based self-evaluation, which may contribute to avoidance, emotional distress, and reduced psychological wellbeing (Tangney and Dearing, 2003). This distinction may have important implications for intervention development, as approaches emphasizing self-compassion and adaptive self-perception may be more beneficial than interventions focused solely on performance-related behavior (Wei et al., 2020).

From a practical perspective, the findings suggest that impostor experiences may be amenable to intervention. Educational strategies that strengthen self-efficacy, encourage adaptive self-reflection, promote self-compassion, and address maladaptive perfectionistic tendencies may help reduce psychological distress associated with IP (Shinawatra et al., 2023; Tangney and Dearing, 2003). Universities may also benefit from fostering supportive learning environments that normalize self-doubt and encourage help-seeking and peer support (Shafran and Mansell, 2001; Hoffman et al., 2002).

It is also important to consider the trait–state nature of the constructs examined. IP, self-efficacy, and perfectionism are often conceptualized as relatively stable traits, yet evidence suggests they can also show state-like variability over time and in response to situational demands (Liu et al., 2023). They may fluctuate with contextual factors such as academic stress, competition, and features of the social environment, including peer comparison and evaluative climates (Zajacova et al., 2005; Damian et al., 2021; Liu et al., 2024). This dynamic perspective implies that students' psychological experiences may vary over time and across situations, highlighting the need for longitudinal research to capture how IP, perfectionism, and self-efficacy co-develop amid changing academic pressures.

## Limitations

Several limitations should be acknowledged. First, the cross-sectional design precludes causal inference, and all observed relationships, including indirect association patterns, should be interpreted as statistical associations rather than directional effects. Second, the indirect association model demonstrated an inconsistent suppression-like pattern, in which the indirect effect had the opposite sign to the total and direct associations. Accordingly, this finding should be considered exploratory and interpreted cautiously, and replication using longitudinal or experimental designs is warranted. Third, the use of convenience and snowball sampling may have introduced selection bias and limited representativeness, as not all eligible students had an equal opportunity to participate. Fourth, although established instruments and rigorous translation procedures were employed, formal construct validation using confirmatory factor analysis was not conducted in the present sample, and the factor structure of the translated measures warrants further examination in future studies. Fifth, perceived academic competition was assessed using a brief two-item measure, which may have limited the breadth of the construct and contributed to conceptual overlap with related psychosocial constructs, particularly sense of belonging. Consequently, the strong association observed between these variables should be interpreted cautiously and warrants further examination using more comprehensive measures in future studies. Finally, the study was conducted at a single institution and relied on self-reported measures, which may limit generalizability and introduce response biases, including social desirability and common method variance.

Future research should employ longitudinal and experimental designs to examine how IP, perfectionism, and self-efficacy evolve over time and influence one another under changing academic demands. Incorporating family characteristics, parental educational attainment, and broader sociocultural factors may provide a more comprehensive understanding of the contextual influences associated with impostor experiences. Multi-institutional and cross-cultural studies are also needed to determine the extent to which these findings generalize across different educational systems and cultural settings.

## Conclusion

The impostor phenomenon (IP) among Saudi university students was associated with higher maladaptive perfectionism and lower self-efficacy, whereas self-efficacy showed stronger positive associations with happiness and a sense of belonging. The findings also identified indirect associations between perfectionism and self-efficacy, as well as several psychological outcomes. Differences observed across academic disciplines suggest that contextual academic factors may influence students' experiences of IP. Given the cross-sectional design and sampling limitations, the findings should be interpreted cautiously. Future longitudinal, multi-institutional, and cross-cultural studies incorporating family and sociocultural factors are needed to better understand the antecedents and developmental trajectories of impostor phenomenon and to inform interventions aimed at promoting students‘ psychological wellbeing.

## Data Availability

The original contributions presented in the study are included in the article/supplementary material, further inquiries can be directed to the corresponding author.
